# Genome-wide identification and characterization of *WOX* gene family in saffron (*Crocus sativus* L.) and their roles in stress response, development and callus formation

**DOI:** 10.3389/fpls.2026.1764909

**Published:** 2026-03-18

**Authors:** Yuanyuan Tao, Jing Li, Jing Chen, Xiaoyuan Xi, Shuhui Yang, Feng Qiu, Xingchang Zhang, Mengqing Feng, Xiaodong Qian, Liqin Li

**Affiliations:** 1 Huzhou Central Hospital, Fifth School of Clinical Medicine of Zhejiang Chinese Medical University, Huzhou, China; 2 Huzhou Central Hospital, Affiliated Central Hospital of Huzhou University, Huzhou, China; 3 Traditional Chinese Medicine (TCM) Key Laboratory Cultivation Base of Zhejiang Province for the Development and Clinical Transformation of Immunomodulatory Drugs, Huzhou, China; 4 School of Pharmacy, Hangzhou Normal University, Hangzhou, China

**Keywords:** *Crocus sativus*, *WOX* genes, SAM, temperature and hormonal stresses, callus formation

## Abstract

**Introduction:**

The shoot apical meristem (SAM) of saffron (*Crocus sativus* L.) plays a critical role in floral transition and development. WUSCHEL-related homeobox (*WOX*) genes are key regulators of stem cell activity and SAM size across diverse species, including *Arabidopsis thaliana, Oryza sativa Japonica, Gossypium hirsutum* L. and the hybrid poplar clone 84K (*Populus alba* × *P. glandulosa*).

**Methods:**

WOX genes were identified and analyzed for phylogeny, gene structure, conserved motifs, cis-regulatory elements, chromosomal location, collinearity and duplication. Expression patterns were examined in different tissues, cold stresses, hormonal stress and callus formation, with key genes validated by real-time quantitative PCR.

**Results:**

In this study, we identified 20 *CsWOX* genes based on the triploid genome of saffron. Intraspecific collinearity analysis revealed two collinear pairs (*CsWOX7/CsWOX5* and *CsWOX13A/CsWOX13B*) in saffron. Furthermore, interspecific synteny was established between *AtWUS* in *A. thaliana* and *CsWUS* in saffron, as well as between saffron and rice (*OsWOX9/CsWOX5, OsWOX9/CsWOX7*, and *OsWOX2/CsWOX3*). Transcriptome analysis was conducted to investigate the expression patterns of *CsWOX* genes in the SAM under temperature and hormonal stresses. Cold treatment (9°C) significantly downregulated *CsWUS* expression during stages S1–S6, while the expression of *CsWOX13B* and *CsWOX4* exhibited a recovery trend during stages S4–S6. Although Gibberellic Acid (GA) and Abscisic Acid (ABA) promoted the differentiation of apical meristems into floral meristems, most *CsWOX* genes remained largely unresponsive or showed only transient responses to these hormones. Additionally, *CsWOX13A/B* expression increased progressively throughout the four stages of stigma development, peaking at stage 4. In corm-derived callus, *CsWUS* and *CsWOX11* were significantly upregulated at the non-shoot-forming (NSF) and shoot-forming (SF) callus stages. Notably, while *CsWOX11* exhibited a similar expression pattern across both corm and stigma sources, *CsWUS* showed a distinct, progressive increase in expression during the NSF and SF stages of stigma-derived callus.

**Discussion:**

This study analyzed the potential functions of different *WOX* genes from multiple perspectives, including temperature stress, hormonal regulation, growth and development, and callus tissue culture. These findings provided a foundation for further investigation into the mechanisms by which candidate *WOX* genes regulate the saffron SAM.

## 1 Introduction

Saffron (*Crocus sativus* L.) is an autotriploid species that propagates exclusively through asexual reproduction. The shoot apical meristem (SAM) stem cells in its aerial part can differentiate into leaves, petals, stigmas and stamens, of which the stigma is of important medicinal value (Li et al., 2025). The *WOX* family is a group of plant specific transcription factors. Each member contains a highly conserved homeodomain (HD) composed of 65–66 amino acid. By regulating target gene expression at the transcriptional level, *WOX* genes regulate virtually every aspect of plant growth and development, including roots, stems, leaves, flowers, fruits, seeds and embryogenesis (Ueda et al., 2011; Romera-Branchat et al., 2013; Costanzo et al., 2014). Among them, *WUS* and *WOX5* are essential for maintaining the balance between stem-cell self-renewal and differentiation in the SAM and root apical meristem (RAM), respectively, ensuring continuous development throughout the plant life cycle (Zhou et al., 2015). As per our knowledge, no studies on the *WOX* gene family in saffron have been reported. Investigating the *WOX* gene family may provide insights into improving the quality and yield of saffron.

The *WOX* gene family is classified into three major clades based on phylogenetic relationships and structural characteristics: the ancient clade (*WOX10*, *WOX13*, and *WOX14*), the intermediate clade (*WOX8*, *WOX9*, *WOX11* and *WOX12*), and the modern clade (*WUS*, *WOX1*-*WOX7*). Members of these distinct clades exhibit significant functional diversification. Within the ancient clade, *AtWOX13* inhibited the regeneration of pluripotent callus by suppressing *WUS* and *STM*, thereby directing cell differentiation toward turgid spherical cells (Ogura et al., 2023). In the intermediate clade, *OsWOX11* recruits the ADA2-GCN5 histone acetyltransferase module to activate downstream targets in the crown root meristem (Zhou et al., 2017), and, together with *CRL1*, maintains cytokinin homeostasis to promote crown root development (Geng et al., 2023). Additionally, *PagWOX11/12a* in poplar confers salt tolerance by directly activating PagCYP736A12, attenuating ROS-induced damage (Wang et al., 2021). In the modern clade, *AtWUS*, the founding member, is expressed in the organizing center (OC) of the SAM, where the WUS–CLV3 negative feedback loop maintains the homeostasis of stem cell pool (Yadav et al., 2011). *AtWUS* cooperates with *LEAFY* (*LFY*) to activate *AGAMOUS* (*AG*) in floral meristems, which in turn terminates *WUS* expression to ensure floral determinacy (Lenhard et al., 2001). *WOX1* and *WOX3* act redundantly to control lateral leaf expansion (Nakata et al., 2012) and are crucial for leaf morphogenesis and margin patterning (Zhang et al., 2020; Xu et al., 2024a). *AtWOX1* temporally modulates the number and size of leaf serrations through the known margin regulators CUC3 and BZR1 (Drieberg, 2025). *AtWOX4* regulates vascular stem cell proliferation via the TDIF peptide signaling (Hirakawa et al., 2010), while *OsWOX4* is cytokinin-inducible and regulates early leaf development (Cheng et al., 2014). Additionally, *WOX5* and *WOX7* regulate root development by sustaining callus proliferation through driving the cell cycle (Zhang et al., 2025). *WOX5* and *WOX7* are specifically expressed in the quiescent center (QC) across species (e.g., Arabidopsis, rice, and maize) and physically interact with PAT1 to modulate transcription of core cell-cycle genes (Yang et al., 2024).

*WOX* genes play crucial regulatory roles in vegetative organogenesis, reproductive organogenesis, tissue regeneration, callus formation and abiotic stress responses across diverse plant species (Zhou et al., 2024). To comprehensively characterize the structural and functional features of the WOX family in saffron, based on saffron genome (Li et al., 2025), we identified *WOX* family members and analyzed their gene structure, *cis*-regulatory elements, duplication events and collinearity. Furthermore, we profiled the expression patterns of *CsWOX* genes across diverse tissues and under abiotic stress conditions. Given that saffron is a high-value medicinal plant whose active compound accumulation is closely linked to its developmental processes, investigating the *WOX* gene family provides crucial insights into the molecular mechanisms governing saffron growth and establishes a foundation for molecular breeding strategies to enhance both the quality and yield of saffron.

## 2 Materials and methods

### 2.1 Identification of *WOX* gene family members in saffron

*WOX* protein sequences of *Arabidopsis thaliana* and *Oryza sativa Japonica* were retrieved from PlantTFDB v4.0 (https://planttfdb.gao-lab.org/) and used as queries for BLAST (E-value < 1e^−5^) against the saffron genome (Li et al., 2025). Additionally, a Hidden Markov Model (HMM) profile search was performed using HMMER 3.0, based on the conserved *homeodomain* of *AtWOX* proteins. The candidate *CsWOX* sequences identified by both BLAST and HMM searches were integrated. Subsequently, the conserved homeodomain was verified using the NCBI Conserved Domain Database (CDD) and SMART. Sequences missing the conserved HB (homeodomain) or redundant sequences were manually removed. Multiple sequence alignment and visualization of conserved motifs were performed using the DNAMAN 9 software.

### 2.2 Phylogenetic analysis and classification of *CsWOX* genes

Phylogenetic analysis was performed on non-redundant *WOX* protein sequences from *C. sativus* and *A. thaliana*. Multiple sequence alignment was performed using MUSCLE with default parameters, and a maximum-likelihood phylogenetic tree was constructed via FastTree based on the JTT model (Price et al., 2009). The resulting tree was visualized using the iTOL (https://itol.embl.de/).

### 2.3 Gene structure, conserved motif and *cis*-regulatory element analyses

Conserved motifs were identified with MEME Suite (https://meme-suite.org), with the maximum number of motifs set to 10. Homeodomain were further validated and annotated using the CDD. Cis-acting regulatory elements in the 2,000 bp promoter regions upstream of the start codon (ATG) were predicted using the PlantCARE database (Lescot et al., 2002). Visualizations of gene structures, conserved motifs and *cis*-regulatory elements were integrated using TBtools-II. Statistical analyses and visualization were performed using GraphPad Prism 10.

### 2.4 Chromosomal location, collinearity and gene duplication analysis of *CsWOX*

The chromosomal distributions of *CsWOX* genes were mapped using TBtools-II. Intraspecific collinearity and duplication events within saffron were identified using MCScanX with default parameters (Wang et al., 2024). The same methodology was applied to identify the duplication types of interspecific syntenic pairs between *C. sativus* and two model species, *A. thaliana* and *O. sativa*. All syntenic relationships and chromosomal localizations were visualized using Circos and TBtools-II.

### 2.5 Gene expression pattern in different tissues and under multiple stresses

Healthy, dormant corms (25 ± 1 g; Huzhou, China) were initially placed at 9 °C and 22 °C. Following dormancy release, the corms were transferred to 25 °C and 17 °C to induce the transition to reproductive growth. Apical buds were collected at six sequential developmental stages (S1-S6): including dormant (S1), dormancy-released (S2), early (S3), mid (S4) and late (S5) floral-bud differentiation, and floral-organ emergence (S6) (Xi et al., 2024). For hormonal treatments, corms (18–22 g; Bozhou, China) were treated with abscisic acid (ABA, 5 mg/L) and gibberellic acid (GA, 5 mg/L) for three consecutive days starting on 17 June and 8 July. The control group received an equal volume of distilled water. Apical buds were then collected at 3, 10, 13, 16 and 19 days after the initial treatment (Chen et al., 2025). Callus culture protocols: corms were subjected to a two-step sterilization procedure (Chib et al., 2020) and cultured on complete MS medium supplemented with 2,4-D (2 mg/L), BAP (1 mg/L) and NAA (1 mg/L). The cultures were maintained at 20 ± 2 °C with 45% relative humidity under a 16/8 h (light/dark) photoperiod. Stigmas explants were directly inoculated under the same conditions. Expression of *CsWOX* genes was quantified as Fragments Per Kilobase of transcript per Million mapped reads (FPKM) and visualized using heatmaps on the Microbial Bioinformatics platform. Statistical evaluation and line chart were performed using GraphPad Prism 10.

### 2.6 Real-time quantitative PCR validation

Total RNA was reverse-transcribed into cDNA using the PrimeScript RT Master Mix (Takara, Japan) from corm and stigma tissues of three distinct stages. Quantitative real-time PCR (qRT-PCR) was performed using TB Green Premix Ex Taq II (Takara, Japan) on an ABI 7500 Real-Time PCR System (Applied Biosystems, USA). All primers utilized in this study were listed in **Supplementary Table 1**. Three biological replicates were performed for each sample. The relative expression levels of the target genes were normalized and calculated using the 2^−ΔΔCT^ method.

## 3 Results

### 3.1 Whole-genome identification of the *WOX* genes family in saffron.

*WOX* proteins are characterized by a conserved homeodomain comprising three α-helices linked by loops and a turn. Conservative residues in Helix 1 include Q, L, and E; in Helix 2, P and L; and in Helix 3, N, V, W, F, Q, N, and R (Yu et al., 2022). By integrating Hidden Markov Model (HMM) profiles and BLASTP searches against the saffron genome database, a total of 20 *CsWOX* genes were identified (**Supplementary Table 2**). Multiple sequence alignment confirmed the high conservation of the characteristic homeodomain across all identified proteins (**Supplementary Figure 1**). Saffron is a triploid species, and our analysis revealed that the 20 *CsWOX* sequences represent nine distinct genes with varying homeologous copy numbers. Specifically, four genes harbored three homeologous copies across the three subgenomes (totaling 12 sequences), while three genes possessed two copies (totaling 6 sequences), and the remaining two genes were identified as single copy sequences. Homeologous copies shared >95% sequence similarity. Only one pair of copies exhibited a 51 amino acid gap (CSa_05_1G0005550/CSa_05_3G0005550), with the remaining regions maintaining high conservation. To ensure comprehensive coverage of key conserved motifs and functional domains for subsequent analyses, we selected one representative sequence for each of the nine *CsWOX* genes (**Supplementary Table 3**). These representative sequences, which exhibited the highest alignment with the NCBI database, were utilized for all subsequent structural, evolutionary, and expression profiling analyses. The amino acid sequences of *CsWOX* genes were subjected to systematic bioinformatic analyses, including molecular weight, theoretical pI, instability index, aliphatic index, and grand average of hydropathicity (GRAVY). As shown in **Supplementary Table 3**, *CsWOX* proteins comprised 163 (*CsWOX*5) to 357 (*CsWOX*9) amino acids, with molecular weights ranging from 18.49 kDa (*CsWOX*5) to 38.31 kDa (*CsWOX*9). Theoretical pI values ranged from 5.69–9.65, aliphatic index from 54.36–79.50, and instability index from 41.18–68.02, classifying them as unstable. CsWOX proteins exhibited negative GRAVY scores, indicating their intrinsic hydrophilicity.

### 3.2 Phylogenetic analysis and subfamily classification of *CsWOX* genes

**Figure 1:**
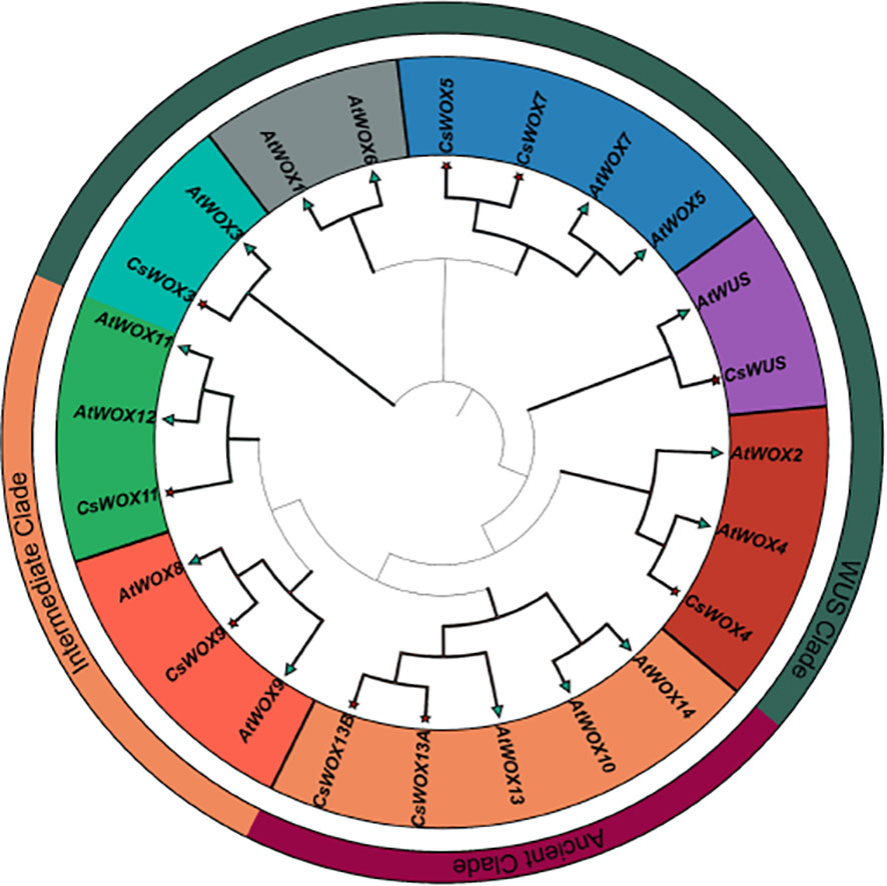
Phylogenetic tree analysis of *AtWOX* (*A. thaliana*) and *CsWOX* (*C. sativus*) proteins. Green triangles and red stars represent *A. thaliana* and *C. sativus*, respectively. They were classified into three major phylogenetic clades: ancient, intermediate, and WUS, which were represented by distinct colors.

To elucidate the evolutionary relationships within the *WOX* gene family between saffron and Arabidopsis, a phylogenetic tree was constructed using WOX protein sequences from *A. thaliana* and *C. sativus* (**Figure 1**). The results demonstrated that *CsWOX* members clustered into three conserved clades, consistent with the classification in Arabidopsis: the ancient clade (*WOX10/13/14*), the intermediate clade (*WOX8/9, WOX11/12*), and the modern/WUS clade (*WOX1–7, WUS*). Notably, orthologs corresponding to *AtWOX1, AtWOX2, AtWOX6, AtWOX8, AtWOX10, AtWOX12*, and *AtWOX14* were not identified in the saffron genome.

### 3.3 Conserved motifs and *cis*−regulatory element analysis of *CsWOX* genes

**Figure 2:**
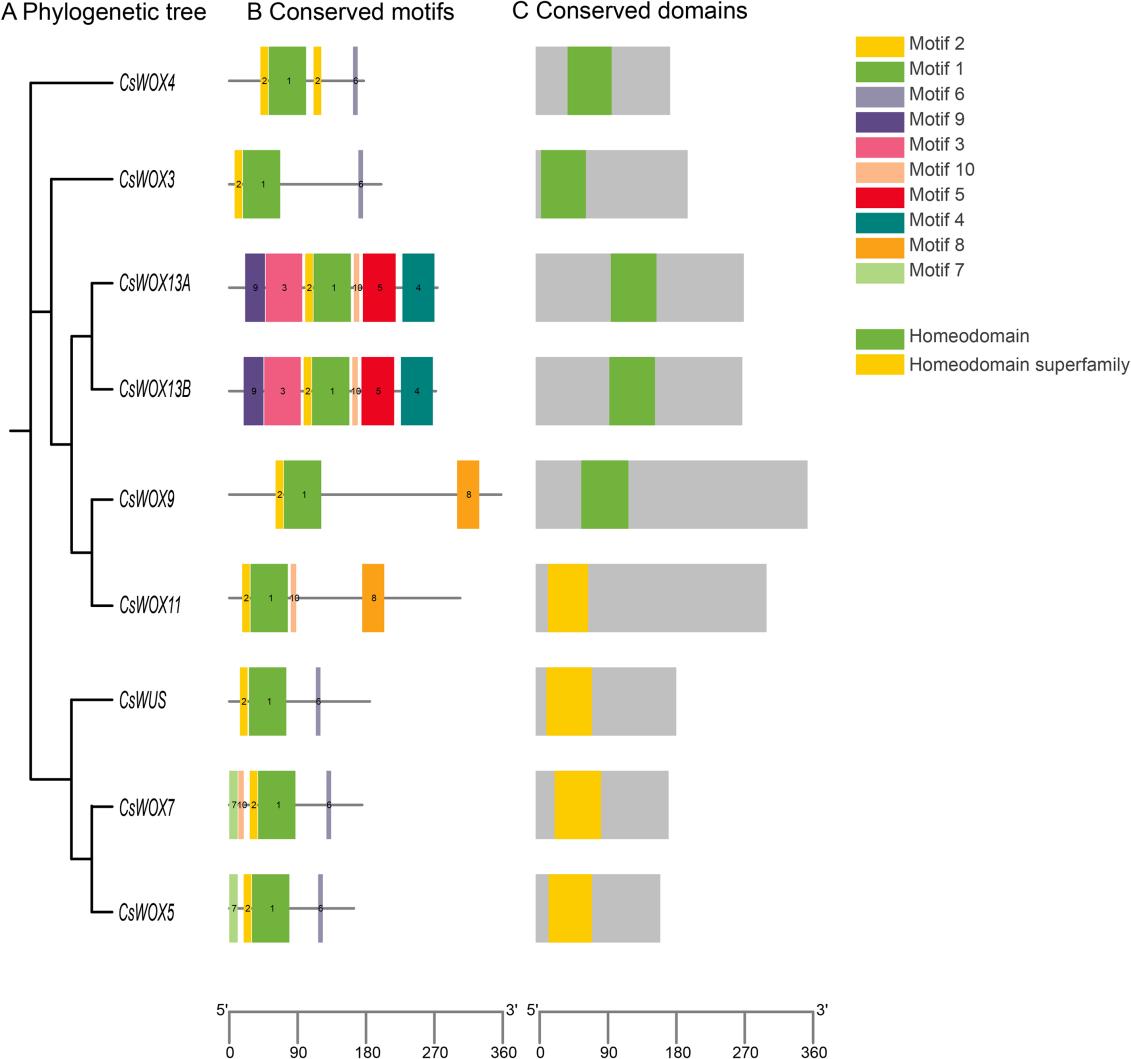
Phylogenetic tree, conserved motifs and conserved domain analysis of *CsWOX* gene family members. **(A)** Phylogenetic tree. **(B)** Distribution of conserved motifs of *CsWOX* proteins. A total of 10 motifs were predicted, represented by colored boxes. The scale bar represents 90 aa. **(C)** Conserved domains of *CsWOX* proteins. The scale bar represents 90 aa.

**Figure 3:**
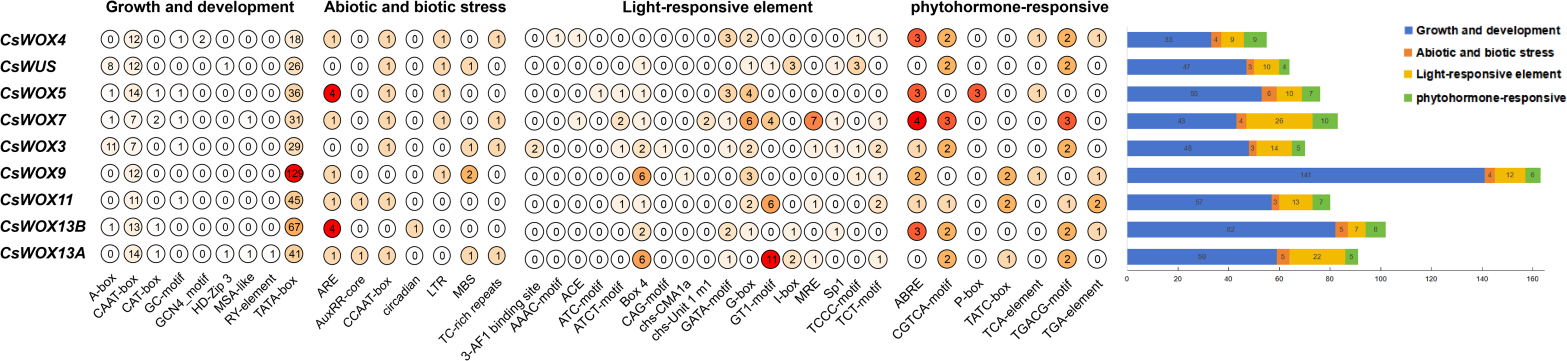
Classification and statistics of predicted CREs on the putative promoter region of *CsWOX* genes. Four major CRE categories were identified: growth/development, stress response (abiotic and biotic), light response, and phytohormone response. Numbers and colors indicate the count of specific elements per gene.

To further investigate the structural and functional diversity of *CsWOX* genes, we analyzed their conserved motifs (**Figure 2**) and *cis*-acting regulatory elements (**Figure 3**). Ten distinct motifs were identified across CsWOX proteins. Different motif combination modes suggested potential functional differences among family members. All *CsWOX* members shared Motif 1 and Motif 2 (**Figure 2B**), which together constituted the characteristic HD (**Figure 2C**). All *CsWOX* genes shared Motif 1 and Motif 2 (**Figure 2B**), which together constituted the characteristic HD (**Figure 2C**). Specifically, *CsWUS* and *CsWOX3* contained Motifs 1, 2, and 6. *CsWOX4* contained an additional Motif 2. *CsWOX5* possessed Motifs 1, 2, 6, and 7, *CsWOX7* carried Motifs 1, 2, 6, 7 and 10. *CsWOX9* contained Motifs 1, 2, and 8, whereas *CsWOX11* additionally included Motif 10. *CsWOX13A/B* lacked Motifs 6, 7, and 8, but retained the other seven motifs. These distinct motif patterns potentially underpin the functional specialization of different *CsWOX* clades.

Analysis of the 2,000 bp promoter regions using the PlantCARE database identified 40 types of CREs across the *CsWOX* genes (**Figure 3**; **Supplementary Tables 4-1**, **4-2**). CREs were classified into four major categories, including abiotic stress responsive (7), hormone responsive (7), light responsive (17) and growth and development related (9). Among development related CREs, HD-Zip3 (vascular/embryo specificity) was present in *CsWUS* and *CsWOX13A*; MSA-like (meristematic root tips/callus) was detected in *CsWOX7* and *CsWOX13A*; the CAT-box (root hair/meristem) occured in *CsWOX7*, *CsWOX5*, *CsWOX13A*/*B*. Regarding stress responses, LTR elements (low-temperature response) were found in *CsWOX4/5/7/9* and *CsWUS*, suggesting their involvement in thermal adaptation. MBS elements (drought inducibility) were present in *CsWOX3/9/13A* and *CsWUS*. Light-responsive CREs (Box 4, G-box) were universally distributed across all *CsWOX* genes promoters. Notably, each *CsWOX* gene contained at least two hormone-responsive elements, including ABRE (ABA response) and MeJA-responsive elements (CGTCA-motif or TGACG-motif), which showed extremely high co-occurrence. This pattern suggested that *CsWOX* genes may be co-regulated by ABA and MeJA signaling pathways (Soma et al., 2021; Xu et al., 2024b). Additionally, GA (TATC-box, P-box), IAA (TGA-element), and SA (TCA-element) responsive elements were differentially distributed among family members, indicating diverse regulatory potentials.

### 3.4 Chromosomal location, collinearity and evolution analysis of *CsWOX* genes

**Figure 4:**
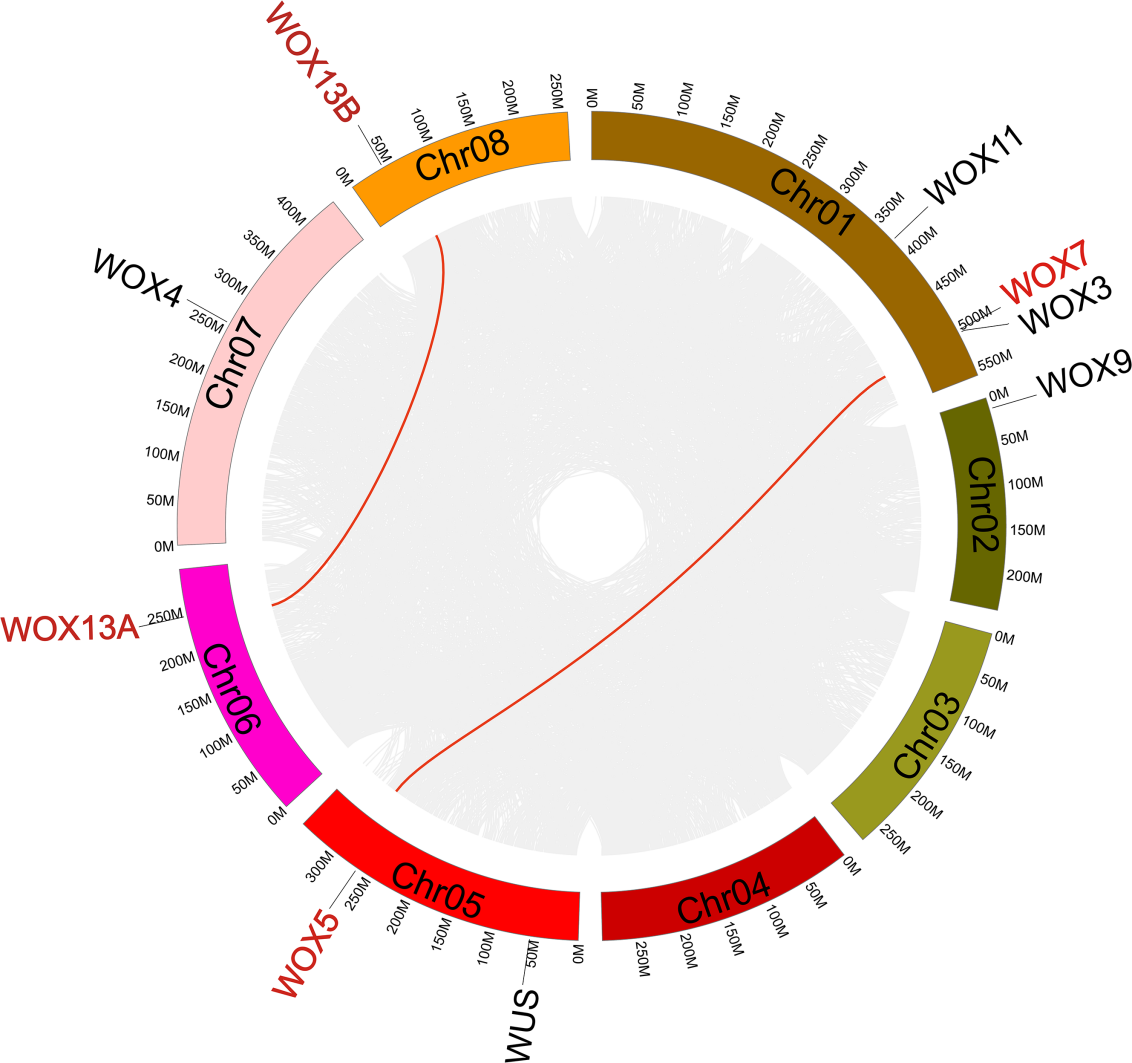
Distribution and colinearity of *CsWOX* genes within the saffron genome. Colinearity genes were highlighted in red. *CsWOX* genes lacking colinearity were marked in black.

The *CsWOX* genes were distributed across all chromosomes except for Chr03 and Chr04. Chr01 and Chr05 each contained three members, whereas the remaining three chromosomes each contained a gene (**Figure 4**). Gene duplication events in the saffron genome were classified into five categories using the MCScanX method, including singleton (1,584 genes), dispersed (5,372 genes), proximal (947 genes), tandem (1,415 genes), and segmental (73,439 genes). The high proportion of segmental events indicated that saffron may have undergone whole genome duplication (WGD) or large segment duplication events. The identification of 5,372 dispersed genes further suggested frequent small segment genomic rearrangements or transposition events, while the paucity of proximal and tandem duplications suggested that local gene amplification was not a primary driver of genome expansion. Among the nine *CsWOX* genes, two WGD gene pairs (*CsWOX13A/CsWOX13B* and *CsWOX7/CsWOX5*) and one dispersed gene pair (*CsWOX11/CsWOX3*) were identified (**Supplementary Table 5**), with no tandem duplication events detected within the family.

**Figure 5:**
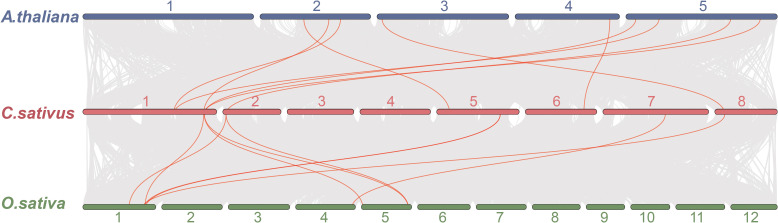
Collinearity analysis of *WOX* genes between *C. sativus* both *A. thaliana* and *O. sativa*. Colinearity genes were highlighted in red.

To further explore the evolutionary history of the *WOX* family, we performed interspecific collinearity analysis between *C. sativus* and two model species, *A. thaliana* and *O. sativa* (**Figure 5**). One segmental duplication pair (At*WUS*/*CsWUS*) and eight dispersed duplication pairs were identified in *A. thaliana* and *C. sativus*. Three segmental duplications pairs (*OsWOX9/CsWOX5*, *OsWOX9/CsWOX7*, and *OsWOX2/CsWOX3*) and six dispersed duplications pairs were identified in *O. sativa* and *C. sativus* (**Supplementary Table 4**). No tandem duplications were found in either comparison. These syntenic relationships suggested that *CsWUS*, *CsWOX5*, *CsWOX7*, and *CsWOX3* are evolutionarily conserved and likely retain functions analogous to their orthologs in *A. thaliana* and *O. sativa*.

### 3.5 Expression patterns of *CsWOX* genes

**Figure 6:**
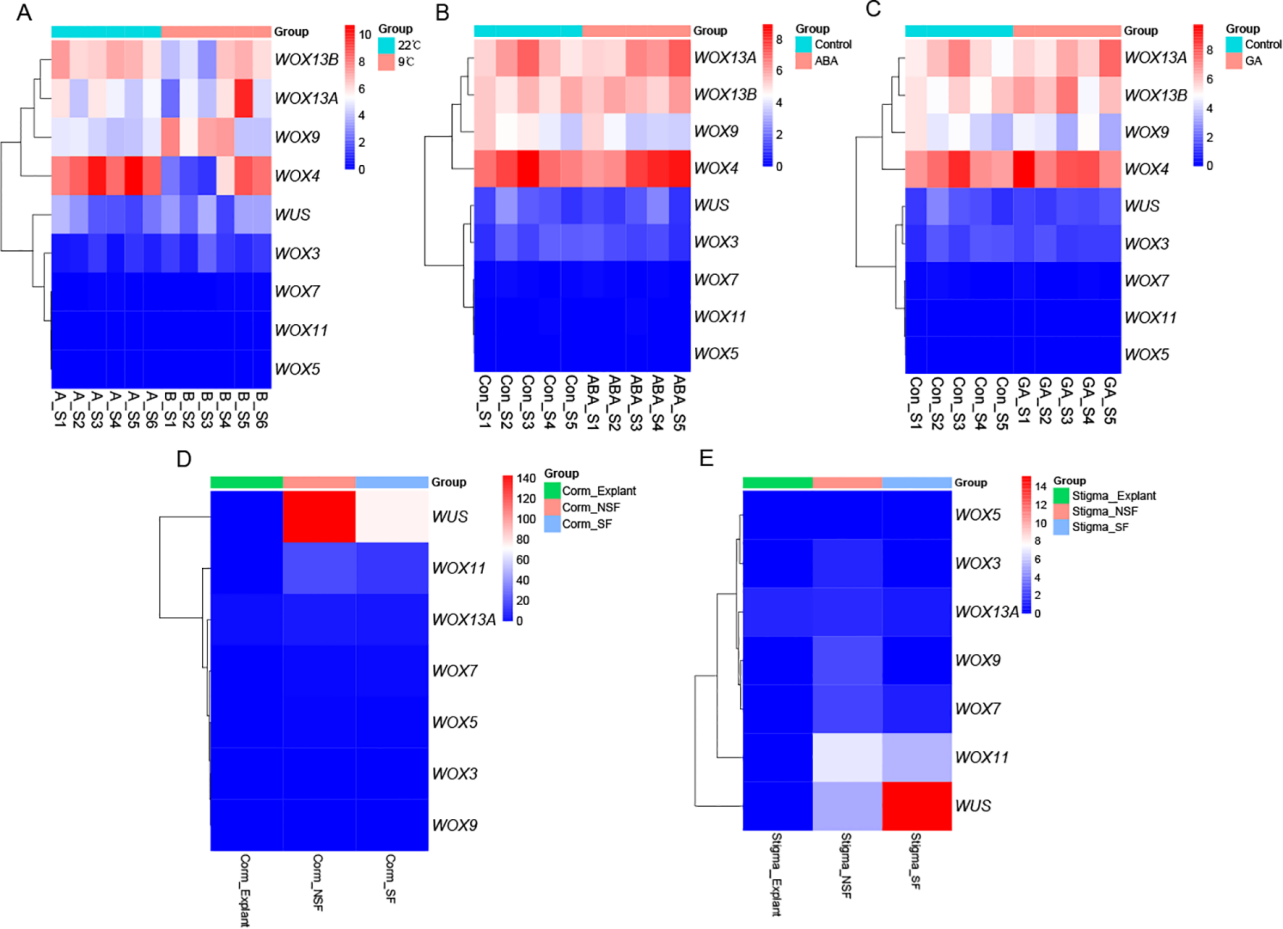
*CsWOX* genes expression patterns. **(A)** *CsWOX* genes expression patterns of SAM with different temperatures treatments. **(B)** The response of *CsWOX* genes in the SAM to ABA. **(C)** The response of *CsWOX* genes in the SAM to GA. **(D)** *CsWOX* genes expression patterns with corm derived callus. **(E)** *CsWOX* genes expression patterns with stigma derived callus. NSF and SF represent the non-shoot-forming and shoot-forming callus stages, respectively. Expression levels are represented by color, with red and blue indicating high and low expression, respectively.

**Figure 7:**
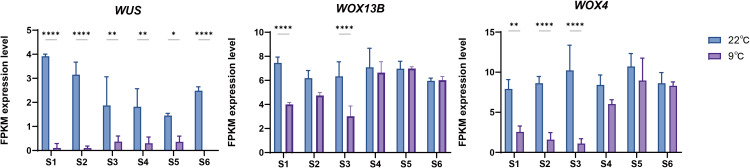
Expression levels of *CsWUS*, *CsWOX13B* and *CsWOX4* in SAM under 9 °C and 22 °C treatment. Asterisks indicate signifcant differences in transcript levels compared with 22 °C treatment (*P < 0.05, **P < 0.01, ****P < 0.0001).

The SAM development of saffron daughter corms progresses through distinct stages, beginning with dormancy and its subsequent release, followed by floral primordium differentiation, and culminating in floral organ maturation. Because the activity of SAM stem cells was influenced by temperature (de Jonge et al., 2016), we investigated *CsWOX* genes expression under different temperatures. As previously reported by our group, corms were stored at 9 °C and 22 °C until dormancy termination. Corms stored at 9 °C exhibited leaf development but failed to differentiate floral buds, whereas those maintained at 22 °C successfully produced both fully developed leaves and floral buds (Xi et al., 2024; Li et al., 2025). To investigate the response of *CsWOX* genes to cold stress, we analyzed RNA-seq data to characterize expression profiles in the SAM under 9 °C and 22 °C treatments (**Figure 6A**). At 22 °C, *CsWUS* showed a gradual downregulation from stages S1 to S6, with a slight increase at S6, whereas the remaining family members exhibited no significant changes. *CsWX13B* and *CsWOX4* were highly expressed at 22 °C, while *CsWOX11*, *CsWOX7*, and *CsWOX5* were nearly silent. Notably, cold treatment (9 °C) significantly suppressed *CsWUS* expression throughout stages S1-S6 compared to the 22 °C. Although *CsWUS* showed slight recovery during stages S4–S5, its transcripts were undetectable by stage S6. *CsWOX*13*B* and *CsWOX4* were markedly downregulated in S1–S3, but recovered during stages S4–S6 (**Figure 7**). These results indicate that cold treatment irreversibly downregulates *WUS* expression, whereas its inhibitory effects on CsWOX13B and *CsWOX4* are reversible.

**Figure 8:**
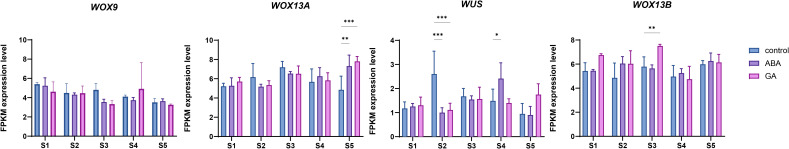
Expression levels of *CsWOX9*, *CsWOX13A*, *CsWUS* and *CsWOX13B* in SAM under ABA and GA treatment. Asterisks indicate signifcant differences in transcript levels compared with control group (*P < 0.05, **P < 0.01, ***P < 0.001).

Our previous studies have indicated that GA and ABA promote the transition of apical meristem differentiation to floral bud differentiation (Chen et al., 2025). During the dormant period, only *CsWOX9* declined gradually in the control group, with no significant trends observed for other *CsWOX* members. ABA treatment specifically upregulated CsWOX13A at S5 and *CsWUS* at S4. GA induced a progressive increase in CsWOX13B at S3 and CsWOX13A at S5. In summary, although GA and ABA promoted the transition to floral meristems, most *CsWOX* genes were either unresponsive or respond only transiently to GA and ABA (**Figures 6B**, **C**, **8**).

**Figure 9:**
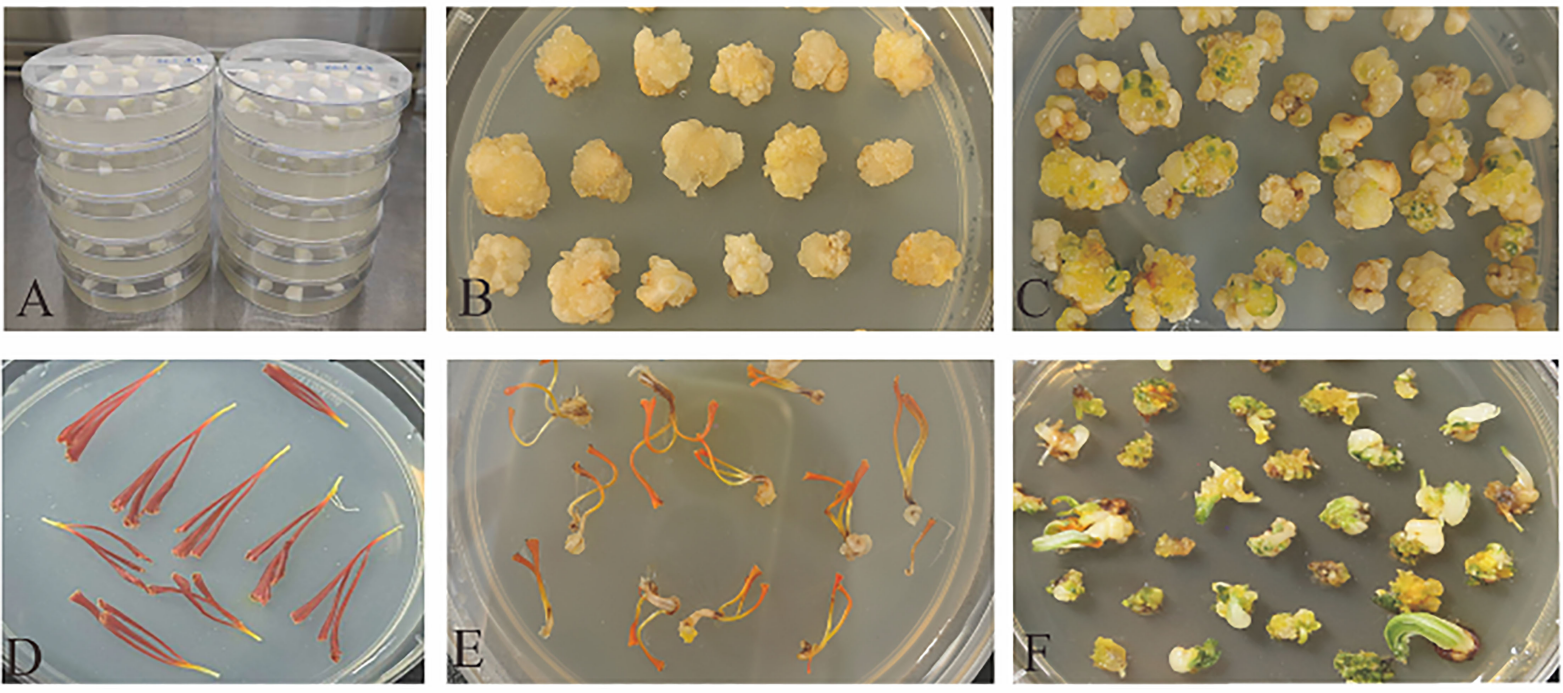
Corm and stigma induced callus formation. **(A)** Corm explants. **(B)** Corm-induced callus (no buds). **(C)** Corm-induced callus (with buds). **(D)** Stigma explants. **(E)** Stigma-induced callus (no buds). **(F)** Stigma-induced callus (with buds).

**Figure 10:**
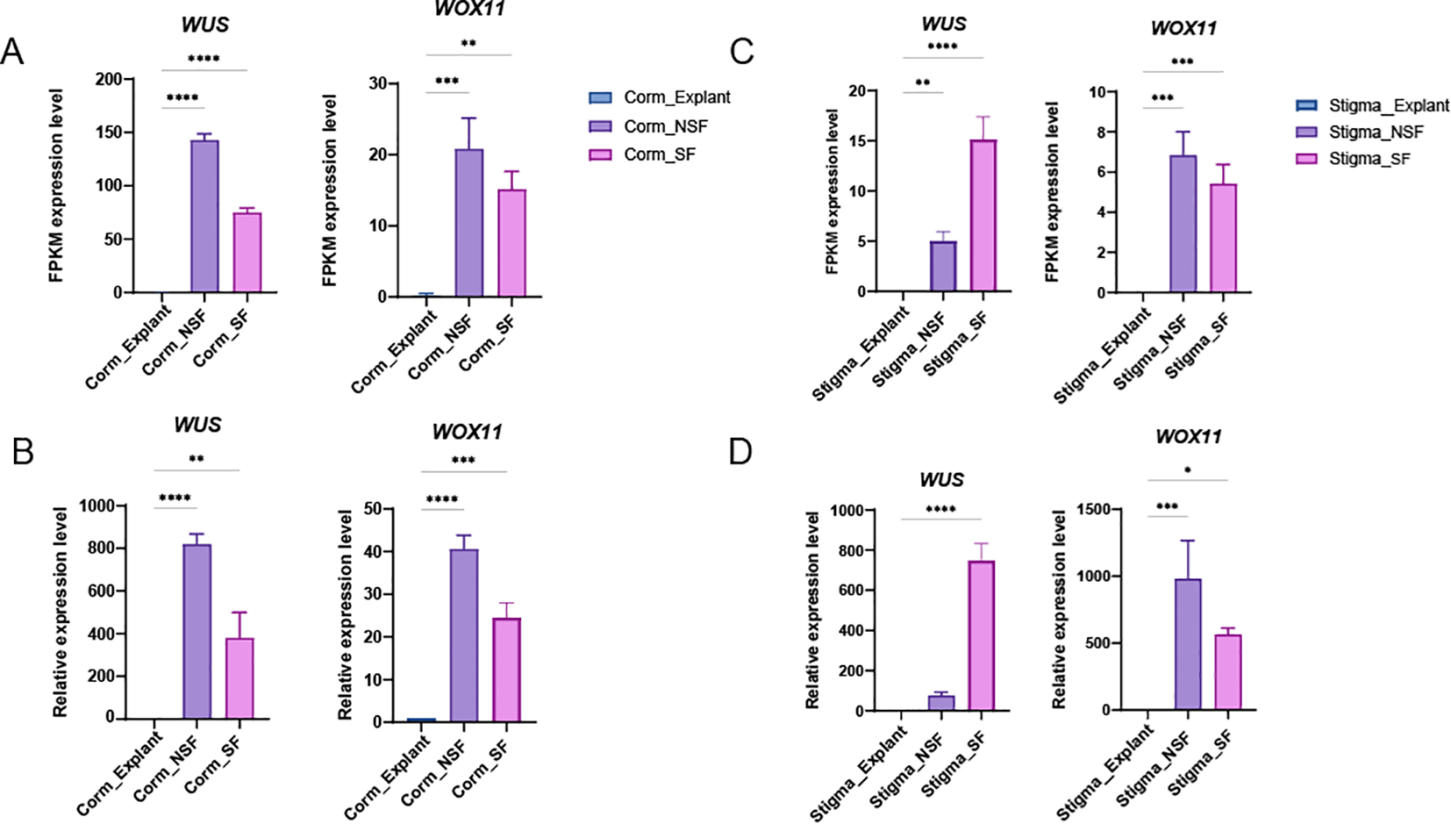
Expression level of *CsWUS* and *CsWOX11* in corm and stigma derived callus and qRT–PCR validation. **(A)** Expression level of *CsWUS* and *CsWOX11* in corm derived callus and **(B)** qRT–PCR validation. **(C)** Expression level of *CsWUS* and *CsWOX11* in stigma derived callus and **(D)** qRT–PCR validation. NSF and SF represent the non-shoot-forming and shoot-forming callus stages, respectively. Asterisks indicate signifcant differences in transcript levels compared with explant group (*P < 0.05, **P < 0.01, ***P < 0.001, ****P < 0.0001).

The *WOX* gene family has been widely utilized to enhance plant tissue and organ regeneration (Jia et al., 2025). Callus induction from corm and stigma explants was shown in **Figure 9**. In corm-derived callus, all nine *CsWOX* genes were nearly undetectable at explant, with only seven *CsWOX* genes recovered at the non-shoot-forming (NSF) and shoot-forming (SF) callus stages (**Figure 6D**). Among these, *WOX3*, *WOX5* and *WOX9* exhibited the lowest expression levels, while *WOX7* and *WOX13A* showed slight expression. In corm-derived callus, *CsWUS* and *CsWOX11* were significantly upregulated at the NSF and SF stages. Notably, while *CsWOX11* exhibited a similar expression pattern across both corm and stigma sources, *CsWUS* showed a distinct, progressive increase in expression during the NSF and SF stages of stigma-derived callus (**Figures 6E**, **10**). These expression profiles were further validated via qRT–PCR (**Figure 10**). These results suggest that *CsWUS* and *CsWOX11* coordinately regulate callus proliferation, and their divergent expression patterns across explant types indicate functional specialization.

We expand the expression profiles of *CsWOX* genes, we examined additional tissues including roots at the flowering stage, four developmental stages of stigma (1–4), and vegetative organs from the propagation stage: mother corm, leaf, and daughter corm (**Supplementary Figure 2**). Results showed that only CsWOX13B, CsWOX13A, CsWOX4 and CsWOX9 were detectable across these tissues, while other members were not detected. CsWOX13B exhibited progressive upregulation during stigma development, peaking at the stigma 4 stage, where expression was significantly higher than in roots. Conversely, *CsWOX13B* expression remained minimal in mother corms, leaves, and daughter corms. In contrast, *CsWOX13A* exhibited marked upregulation in roots, mother corms, leaves, and daughter corms. Although its overall abundance in stigmas was relatively lower, *CsWOX13A* still demonstrated a significant upward trend from stigma 1 to stigma 4 during stigma development. *CsWOX4* and *CsWOX9* were expressed in flowering roots and stigmas 1, with their expression in the stigma 1 significantly exceeding that observed in roots.

## 4 Discussion

The *WOX* gene family is crucial for maintaining the balance between stem cell self-renewal and differentiation, ensuring continuous plant growth throughout the life cycle (Dolzblasz et al., 2016). In *C. sativus*, we identified 20 *WOX* genes. Several genes harbored three homeologous copies across the three subgenomes, while others exhibit gene loss or pseudogenization. This phenomenon may stem from specific subgenome gene deletions, pseudogenization, or regional assembly gaps. Representative copies with the most complete ORFs and conserved domains were selected from each homeologous group to minimize redundancy and ensure reliable evolutionary reconstruction. Transcriptome analysis confirmed that these highly similar copies (>95% identity) displayed nearly identical expression profiles across developmental stages (**Supplementary Figures 3**–**8**), indicating functional redundancy and validating our representative-based approach.

Phylogenetic analysis revealed that *CsWOX* genes were distributed across the ancient, intermediate, and *WUS* clades, consistent with *Arabidopsis* (**Figure 1**). However, orthologs corresponding to *AtWOX1, AtWOX2, AtWOX6, AtWOX8, AtWOX10, AtWOX12*, and *AtWOX14* were not identified in the saffron genome. The absence of these orthologs may reflect functional redundancy among clade members or evolutionary gene loss. For example, *AtWOX5* and *AtWOX7* are the closest homologs (Yang et al., 2024). *AtWOX11* and *AtWOX12* function redundantly (Wang et al., 2021). *WOX4* acts redundantly with *WOX14* downstream of the PXY signaling pathway to promote vascular cell proliferation (Etchells et al., 2013). *AtWOX10* may be a pseudogene, as no transcripts have ever been detected (Deveaux et al., 2008). Intriguingly, two *WOX13* copies were presented in saffron, which also observed in *Akebia trifoliata* and *Gossypium raimondii* (Yang et al., 2017; Han et al., 2024). Genes with analogous functions may have been eliminated during evolution (Chen et al., 2009).

Collinearity analysis revealed that 88.74% of saffron genes originated from WGD and segmental duplications (**Supplementary Table 5**). This likely stemmed from two critical whole-genome triploidy events during plant evolution (Xu et al., 2024c). Within the *CsWOX* family, *CsWOX7/CsWOX5* and *CsWOX13A/CsWOX13B* were identified as WGD-derived duplicates, indicating that saffron has experienced a WGD event. Additionally, the collinear relationships of OsWOX9/CsWOX5, OsWOX9/CsWOX7, OsWOX2/CsWOX3 and AtWUS/CsWUS further confirmed the conserved homology of *WOX* family genes among *C. sativus*, *A. thaliana* and *O. sativa*.

The SAM in bulbous species like *Lilium candidum* and *C. sativus* is highly sensitive to low temperatures that suppress bolting and flowering (Mazor et al., 2021). Our previous study revealed that cold exposure (9 °C) during corm dormancy enabled leaf differentiation but entirely suppressed floral organ formation (Li et al., 2025). Transcriptome analysis of the SAM revealed that cold treatment significantly suppressed CsWUS expression from stages S1 to S6. Similarly, in tomato, *SlWUS* expression decreases significantly under cold stress. Enhanced SlWUS expression is associated with the MF formation (Wu et al., 2023). This suggests that the low-temperature mediated inhibition of CsWUS likely disrupts the stem cell niche required for floral organ differentiation, thereby terminating flowering. Intriguingly, while *CsWOX13B* and *CsWOX4* were initially suppressed by cold (S1–S3), their expression levels recovered during later stages (S4–S6). Given that *WOX4* and *WOX13* are involved in leaf primordia vascular development and grafting success (Ji et al., 2010; Yue et al., 2020), their resilient expression profiles may explain why leaf development remains unaffected by cold stress while floral organs fail to form.

During corm dormancy, we exogenously applied ABA and GA to SAM of saffron. GA significantly enhanced floral primordium differentiation, ABA produced positive but non-significant effect, whereas CK showed no promotion at all. Nevertheless, no significant trend changes were observed in *CsWOX* genes, with almost no variation detected. GA and ABA drive floral transition and floral organ primordia differentiation in *Orchid Cymbidium Sinense* by activating the expression of floral meristem specific genes AP1 (Ahmad et al., 2024). CK and *WUS* genes synergistically interact through complex feedback loops in SAMs, ensuring the balance between stem cell homeostasis and organogenesis (Lee et al., 2016). This suggest that *CsWOX* genes may maintaining stem cell homeostasis and remain less responsive to hormone induced floral primordia initiation during dormancy.

Callus culture is valued as a core tool in agriculture and horticulture (Guo et al., 2015; Efferth, 2019), enabling the development of transgenic lines with enhanced tolerance to abiotic and biotic stresses (Benhayyim and Goffer, 1989; Chavarriaga-Aguirre et al., 2016), and improved yield and nutritional quality (Bahgat et al., 2009). Studies have shown that AtWOX11 is rapidly activated under high auxin conditions to initiate callus formation via LBD16 and WOX5/7 (Sheng et al., 2017), whereas cytokinin signaling triggers WUS expression through chromatin remodeling and HD-ZIP III–type-B ARR complex formation to establish shoot primordia (Zhang et al., 2017; Wan et al., 2023; Liu et al., 2025). Our results suggest that *CsWUS* and *CsWOX11* were concurrently and significantly upregulated at the NSF stage and downregulated at the SF stage in corm-derived callus. Notably, while *CsWOX11* exhibited similar expression pattern both corm and stigma callus, *CsWUS* showed a distinct, progressive increase during the NSF and SF stages of stigma-derived callus. These results indicate that the roles of *CsWUS* and *CsWOX11* in callus formation and differentiation (Ikeda and Ohme-Takagi, 2014) are conserved across multiple model plants, including *Arabidopsis thaliana*, *Gossypium hirsutum* L. and the hybrid poplar clone 84K (*Populus alba* × *P. glandulosa*) (Liu et al., 2014, 2018; Wei et al., 2022). Additionally, variations in the expression of *CsWOX11* and *CsWUS* across different plant species and explant types may reflect distinct regeneration pathways, regulatory variations within a conserved pathway, or species-specific expression programs. In this study, callus developmental stages were defined solely by morphological criteria, which may not fully capture the underlying molecular heterogeneity. Comprehensive characterization of callus development requires integrating morphological, molecular, and functional approaches. Future studies are needed to determine whether *CsWOX11* and *CsWUS* act coordinately or sequentially in the saffron regeneration pathway.

Following flowering, saffron transitions to a vegetative phase where mother corms supply carbon to developing daughter corms (Jose-Santhi et al., 2024). Most *CsWOX* genes were exhibited either no expression or low expression levels during this stage, similar to the expression patterns of *SsuWOX* genes (Jia et al., 2025). Notably, *CsWOX13B* expression increased progressively throughout the four stages of stigma development, peaking at stage stigma 4. *GhWOX13* was highly expressed at 10 and 15 DPA (days post anthesis) and involved in phytohormone-mediated fiber development in cotton (He et al., 2019). Similarly, *AtWOX13* plays important role in controlling the medio lateral pattern of the fruit development, which is fundamental for seed dispersal (Romera-Branchat et al., 2013). This indicated the functional diversity of *CsWOX13*.

## 5 Conclusion

Based on the saffron genome, the study identified 20 CsWOX gene copies. Phylogenetic analysis revealed that the saffron WOX gene family can be classified into three conserved clades: the ancient, intermediate, and WUS clades. Analyses of conserved motif and cis−regulatory elements indicated that genes within the same clade exhibit similar structures and functions. Collinearity analysis identified two WGD derived duplicate pairs (CsWOX7/5 and CsWOX13A/B). Interspecific synteny analysis further confirmed the conserved homology of WOX family genes among Saffron, Arabidopsis, and Rice. During daughter corm development following saffron flowering, most *CsWOX* genes exhibited low or undetectable expression in roots, mother corms, leaves, and daughter corms. Notably, CsWOX13A and CsWOX13B showed progressively increasing expression concomitant with stigma maturation, peaking at stage stigma 4. Crucially, the downregulation of CsWUS in the SAM following cold treatment was identified as a potential molecular trigger for floral primordia abortion. No significant expression changes were observed in CsWOX genes after ABA or GA treatment of SAM. During callus induction from corm and stigma explants, both CsWOX11 and *CsWUS* were significantly upregulated in the NSF and SF callus, supporting their conserved roles in cell dedifferentiation and regeneration. In summary, this study provides a systematic analysis of the WOX gene family in saffron, offering valuable insights for subsequent functional studies of CsWOX genes and establishing a theoretical foundation for practical applications in saffron meristem regulation, callus culture optimization, and molecular breeding.

## Data Availability

The data presented in the study are deposited in the Figshare repository (https://figshare.com/), accession number 10.6084/m9.figshare.31623259.

## References

[B1] Ahmad S. Lu C. Gao J. Wei Y. Xie Q. Jin J. . (2024). Integrated proteomic, transcriptomic, and metabolomic profiling reveals that the gibberellin–abscisic acid hub runs flower development in the Chinese orchid Cymbidium sinense. Hortic. Res. 11, uhae073. doi: 10.1093/hr/uhae073. PMID: 38738212 PMC11088716

[B2] Bahgat S. Shabban O. A. El-Shihy O. Lightfoot D. A. El-Shemy H. A. (2009). Establishment of the regeneration system for Vicia faba L. Curr. Issues Mol. Biol. 11, I47–i54. doi: 10.21775/cimb.011.i47. PMID: 19193964

[B3] Benhayyim G. Goffer Y. (1989). Plantlet regeneration from a NaCl-selected salt-tolerant callus culture of Shamouti orange (Citrus sinensis L. Osbeck). Plant Cell Rep. 7, 680–683. doi: 10.1007/BF00272060. PMID: 24240461

[B4] Chavarriaga-Aguirre P. Brand A. Medina A. Prias M. Escobar R. Martinez J. . (2016). The potential of using biotechnology to improve cassava: a review. In Vitro Cell. Dev. Biology-Plant 52, 461–478. doi: 10.1007/s11627-016-9776-3. PMID: 27818605 PMC5071364

[B5] Chen J. Yang S. Qian X. Zhang X. Tao Y. Li J. . (2025). The impact of phytohormones on the number and quality of flowers in Crocus sativus. BMC Plant Biol. 25, 683. doi: 10.1186/s12870-025-06712-6. PMID: 40410691 PMC12100840

[B6] Chen S. K. Kurdyukov S. Kereszt A. Wang X. D. Gresshoff P. M. Rose R. J. (2009). The association of homeobox gene expression with stem cell formation and morphogenesis in cultured Medicago truncatula. Planta 230, 827–840. doi: 10.1007/s00425-009-0988-1. PMID: 19639337 PMC2729979

[B7] Cheng S. Huang Y. Zhu N. Zhao Y. (2014). The rice WUSCHEL-related homeobox genes are involved in reproductive organ development, hormone signaling and abiotic stress response. Gene 549, 266–274. doi: 10.1016/j.gene.2014.08.003. PMID: 25106855

[B8] Chib S. Thangaraj A. Kaul S. Dhar M. K. Kaul T. (2020). Development of a system for efficient callus production, somatic embryogenesis and gene editing using CRISPR/Cas9 in Saffron (Crocus sativus L.). Plant Methods 16, 47. doi: 10.1186/s13007-020-00589-2. PMID: 32280363 PMC7137501

[B9] Costanzo E. Trehin C. Vandenbussche M. (2014). The role of WOX genes in flower development. Ann. Bot. 114, 1545–1553. doi: 10.1093/aob/mcu123. PMID: 24973416 PMC4204783

[B10] de Jonge J. Kodde J. Severing E. I. Bonnema G. Angenent G. C. Immink R. G. H. . (2016). Low temperature affects stem cell maintenance in Brassica oleracea seedlings. Front. Plant Sci. 7. doi: 10.3389/fpls.2016.00800. PMID: 27375654 PMC4896912

[B11] Deveaux Y. Toffano-Nioche C. Claisse G. Thareau V. Morin H. Laufs P. . (2008). Genes of the most conserved WOX clade in plants affect root and flower development in Arabidopsis. BMC Evol. Biol. 8, 291. doi: 10.1186/1471-2148-8-291. PMID: 18950478 PMC2584047

[B12] Dolzblasz A. Nardmann J. Clerici E. Causier B. van der Graaff E. Chen J. . (2016). Stem cell regulation by Arabidopsis WOX genes. Mol. Plant 9, 1028–1039. doi: 10.1016/j.molp.2016.04.007. PMID: 27109605

[B13] Drieberg H. (2025). WOX out, those teeth are sharp! J. Exp. Bot. 76, 195–197. doi: 10.1093/jxb/erae485. PMID: 39786161 PMC11714750

[B14] Efferth T. (2019). Biotechnology applications of plant callus cultures. Engineering 5, 50–59. doi: 10.1016/j.eng.2018.11.006. PMID: 41781244

[B15] Etchells J. P. Provost C. M. Mishra L. Turner S. R. (2013). WOX4 and WOX14 act downstream of the PXY receptor kinase to regulate plant vascular proliferation independently of any role in vascular organisation. Development 140, 2224–2234. doi: 10.1242/dev.091314. PMID: 23578929 PMC3912870

[B16] Geng L. Li Q. Jiao L. Xiang Y. Deng Q. Zhou D.-X. . (2023). WOX11 and CRL1 act synergistically to promote crown root development by maintaining cytokinin homeostasis in rice. New Phytol. 237, 204–216. doi: 10.1111/nph.18522. PMID: 36208055

[B17] Guo Y. Wiegert-Rininger K. E. Vallejo V. A. Barry C. S. Warner R. M. (2015). Transcriptome-enabled marker discovery and mapping of plastochron-related genes in Petunia spp. BMC Genomics 16, 726. doi: 10.1186/s12864-015-1931-4. PMID: 26400485 PMC4581106

[B18] Han N. Li F. Zhu H. Li T. Wang X. Li T. . (2024). Comprehensive analysis of WOX transcription factors provide insight into genes related to the regulation of unisexual flowers development in Akebia trifoliata. Int. J. Biol. Macromol. 260, 129486. doi: 10.1016/j.ijbiomac.2024.129486. PMID: 38237833

[B19] He P. Zhang Y. Liu H. Yuan Y. Wang C. Yu J. . (2019). Comprehensive analysis of WOX genes uncovers that WOX13 is involved in phytohormone-mediated fiber development in cotton. BMC Plant Biol. 19, 312. doi: 10.1186/s12870-019-1892-x. PMID: 31307379 PMC6632001

[B20] Hirakawa Y. Kondo Y. Fukuda H. (2010). TDIF peptide signaling regulates vascular stem cell proliferation via the WOX4 homeobox gene in Arabidopsis. Plant Cell 22, 2618–2629. doi: 10.1105/tpc.110.076083. PMID: 20729381 PMC2947162

[B21] Ikeda M. Ohme-Takagi M. (2014). TCPs, WUSs, and WINDs: families of transcription factors that regulate shoot meristem formation, stem cell maintenance, and somatic cell differentiation. Front. Plant Sci. 5. doi: 10.3389/fpls.2014.00427. PMID: 25232356 PMC4153042

[B22] Ji J. Strable J. Shimizu R. Koenig D. Sinha N. Scanlon M. J. (2010). WOX4 promotes procambial development. Plant Physiol. 152, 1346–1356. doi: 10.1104/pp.109.149641. PMID: 20044450 PMC2832261

[B23] Jia Y. Lin Z. He H. Zhou Z. Gao K. Du K. . (2025). Comprehensive analysis and identification of the WOX gene family in Schima superba and the key gene SsuWOX1 for enhancing callus regeneration capacity. BMC Plant Biol. 25, 367. doi: 10.1186/s12870-025-06377-1. PMID: 40114040 PMC11924843

[B24] Jose-Santhi J. Sheikh F. R. Kalia D. Sood R. Kumar R. Acharya V. . (2024). Transcriptional dynamics in source-sink tissues identifies molecular factors regulating the corm development process in saffron (Crocus sativus L.). Physiol. Plant 176, e14285. doi: 10.1111/ppl.14285. PMID: 38606764

[B25] Lee D.-K. Parrott D. L. Adhikari E. Fraser N. Sieburth L. E. (2016). The mobile bypass signal arrests shoot growth by disrupting shoot apical meristem maintenance, cytokinin signaling, and WUS transcription factor expression. Plant Physiol. 171, 2178–2190. doi: 10.1104/pp.16.00474. PMID: 27208247 PMC4936579

[B26] Lenhard M. Bohnert A. Jürgens G. Laux T. (2001). Termination of stem cell maintenance in Arabidopsis floral meristems by interactions between WUSCHEL and AGAMOUS. Cell. 105, 805–814. doi: 10.1016/s0092-8674(01)00390-7. PMID: 11440722

[B27] Lescot M. Déhais P. Thijs G. Marchal K. Moreau Y. Van de Peer Y. . (2002). PlantCARE, a database of plant cis-acting regulatory elements and a portal to tools for in silico analysis of promoter sequences. Nucleic Acids Res. 30, 325–327. doi: 10.1093/nar/30.1.325. PMID: 11752327 PMC99092

[B28] Li L. He J. Qian X. Xi X. Li J. Chen J. . (2025). A resource for improving the quality and yield of Crocus sativus stigma. Ind. Crops Prod. 237, 122141. doi: 10.1016/j.indcrop.2025.122141. PMID: 41781244

[B29] Liu J. Sheng L. Xu Y. Li J. Yang Z. Huang H. . (2014). WOX11 and 12 are involved in the first-step cell fate transition during de novo root organogenesis in Arabidopsis. Plant Cell 26, 1081–1093. doi: 10.1105/tpc.114.122887. PMID: 24642937 PMC4001370

[B30] Liu L. Shu Y. F. Wang Y. Liu M. Y. Xu S. X. Lu X. F. . (2025). The pan genome analysis of WOX gene family in apple and the two sides of MdWUS-1 in promoting leaf-borne shoot. Hortic. Res. 12, 13. doi: 10.1093/hr/uhaf117. PMID: 40661132 PMC12258036

[B31] Liu B. Zhang J. Yang Z. Matsui A. Seki M. Li S. . (2018). PtWOX11 acts as master regulator conducting the expression of key transcription factors to induce de novo shoot organogenesis in poplar. Plant Mol. Biol. 98, 389–406. doi: 10.1007/s11103-018-0786-x. PMID: 30324253

[B32] Mazor I. Weingarten-Kenan E. Zaccai M. (2021). The developmental stage of the shoot apical meristem affects the response of Lilium candidum bulbs to low temperature. Sci. Hortic. 276, 11. doi: 10.1016/j.scienta.2020.109766. PMID: 41781244

[B33] Nakata M. Matsumoto N. Tsugeki R. Rikirsch E. Laux T. Okada K. (2012). Roles of the middle domain-specific WUSCHEL-RELATED HOMEOBOX genes in early development of leaves in Arabidopsis. Plant Cell 24, 519–535. doi: 10.1105/tpc.111.092858. PMID: 22374393 PMC3315230

[B34] Ogura N. Sasagawa Y. Ito T. Tameshige T. Kawai S. Sano M. . (2023). WUSCHEL-RELATED HOMEOBOX 13 suppresses de novo shoot regeneration via cell fate control of pluripotent callus. Sci. Adv. 9, eadg6983. doi: 10.1126/sciadv.adg6983. PMID: 37418524 PMC10328406

[B35] Price M. N. Dehal P. S. Arkin A. P. (2009). FastTree: computing large minimum evolution trees with profiles instead of a distance matrix. Mol. Biol. Evol. 26, 1641–1650. doi: 10.1093/molbev/msp077. PMID: 19377059 PMC2693737

[B36] Romera-Branchat M. Ripoll J. J. Yanofsky M. F. Pelaz S. (2013). The WOX13 homeobox gene promotes replum formation in the Arabidopsis thaliana fruit. Plant J. 73, 37–49. doi: 10.1111/tpj.12010. PMID: 22946675

[B37] Sheng L. Hu X. Du Y. Zhang G. Huang H. Scheres B. . (2017). Non-canonical WOX11-mediated root branching contributes to plasticity in Arabidopsis root system architecture. Development 144, 3126–3133. doi: 10.1242/dev.152132. PMID: 28743799 PMC5611959

[B38] Soma F. Takahashi F. Yamaguchi-Shinozaki K. Shinozaki K. (2021). Cellular phosphorylation signaling and gene expression in drought stress responses: ABA-dependent and ABA-independent regulatory systems. Plants 10, 756. doi: 10.3390/plants10040756. PMID: 33924307 PMC8068880

[B39] Ueda M. Zhang Z. Laux T. (2011). Transcriptional activation of Arabidopsis axis patterning genes WOX8/9 links zygote polarity to embryo development. Dev. Cell 20, 264–270. doi: 10.1016/j.devcel.2011.01.009. PMID: 21316593

[B40] Wan Q. Zhai N. Xie D. Liu W. Xu L. (2023). WOX11: the founder of plant organ regeneration. Cell Regener. 12, 1. doi: 10.1186/s13619-022-00140-9. PMID: 36596978 PMC9810776

[B41] Wang Y. Tang H. Wang X. Sun Y. Joseph P. V. Paterson A. H. (2024). Detection of colinear blocks and synteny and evolutionary analyses based on utilization of MCScanX. Nat. Protoc. 19, 2206–2229. doi: 10.1038/s41596-024-00968-2. PMID: 38491145

[B42] Wang L.-Q. Wen S.-S. Wang R. Wang C. Gao B. Lu M.-Z. (2021). PagWOX11/12a activates PagCYP736A12 gene that facilitates salt tolerance in poplar. Plant Biotechnol. J. 19, 2249–2260. doi: 10.1111/pbi.13653. PMID: 34170605 PMC8541782

[B43] Wei X. Geng M. Li J. Duan H. Li F. Ge X. (2022). GhWOX11 and GhWOX12 promote cell fate specification during embryogenesis. Ind. Crops Prod. 184, 115031. doi: 10.1016/j.indcrop.2022.115031. PMID: 41781244

[B44] Wu J. Q. Sun W. R. Sun C. Xu C. M. Li S. Li P. X. . (2023). Cold stress induces malformed tomato fruits by breaking the feedback loops of stem cell regulation in floral meristem. New Phytol. 237, 2268–2283. doi: 10.1111/nph.18699. PMID: 36564973

[B45] Xi X. Li J. Song J. Qian X. Xu X. Feng M. . (2024). CsERECTA alternative splicing regulates the flowering numbers depending on temperature in Crocus sativus L. Ind. Crops Prod. 218, 118971. doi: 10.1016/j.indcrop.2024.118971. PMID: 41781244

[B46] Xu Z. Chen S. Wang Y. Tian Y. Wang X. Xin T. . (2024c). Crocus genome reveals the evolutionary origin of crocin biosynthesis. Acta Pharm. Sin. B. 14, 1878–1891. doi: 10.1016/j.apsb.2023.12.013. PMID: 38572115 PMC10985130

[B47] Xu L. Fang N. Lu T. Tameshige T. Nakata M. T. Jiang Y. . (2024a). WOX1 controls leaf serration development via temporally restricting BRASSINAZOLE RESISTANT 1 and CUP SHAPED COTYLEDON 3 expression in Arabidopsis. J. Exp. Bot. 76, 478–492. doi: 10.1093/jxb/erae443. PMID: 39478336 PMC11714755

[B48] Xu L. Zhu X. Yi F. Liu Y. Sod B. Li M. . (2024b). A genome-wide study of the lipoxygenase gene families in Medicago truncatula and Medicago sativa reveals that MtLOX24 participates in the methyl jasmonate response. BMC Genomics 25, 195. doi: 10.1186/s12864-024-10071-1. PMID: 38373903 PMC10875803

[B49] Yadav R. K. Perales M. Gruel J. Girke T. Jonsson H. Reddy G. V. (2011). WUSCHEL protein movement mediates stem cell homeostasis in the Arabidopsis shoot apex. Genes Dev. 25, 2025–2030. doi: 10.1101/gad.17258511. PMID: 21979915 PMC3197201

[B50] Yang Z. Gong Q. Qin W. Yang Z. Cheng Y. Lu L. . (2017). Genome-wide analysis of WOX genes in upland cotton and their expression pattern under different stresses. BMC Plant Biol. 17, 113. doi: 10.1186/s12870-017-1065-8. PMID: 28683794 PMC5501002

[B51] Yang Y. Liu C. Yu Y. Ran G. Zhai N. Pi L. (2024). *WUSCHEL RELATED HOMEOBOX5* and *7* maintain callus development by promoting cell division in *Arabidopsis*. Plant Sci. 346, 112133. doi: 10.1016/j.plantsci.2024.112133. PMID: 38795752

[B52] Yu Y. Yang M. Liu X. Xia Y. Hu R. Xia Q. . (2022). Genome-wide analysis of the WOX gene family and the role of EjWUSa in regulating flowering in loquat (Eriobotrya japonica). Front. Plant Sci. 13. doi: 10.3389/fpls.2022.1024515. PMID: 36407616 PMC9669421

[B53] Yue J. Yang H. Yang S. Wang J. (2020). TDIF overexpression in poplars retards internodal elongation and enhances leaf venation through interaction with other phytohormones. Tree Physiol. 40, 60–72. doi: 10.1093/treephys/tpz126. PMID: 31860723

[B54] Zhang N. Bitterli P. Oluoch P. Hermann M. Aichinger E. Groot E. P. . (2025). Deciphering the molecular logic of WOX5 function in the root stem cell organizer. EMBO J. 44, 281–303. doi: 10.1038/s44318-024-00302-2. PMID: 39558109 PMC11696986

[B55] Zhang T.-Q. Lian H. Zhou C.-M. Xu L. Jiao Y. Wang J.-W. (2017). A two-step model for de novo activation of WUSCHEL during plant shoot regeneration. Plant Cell 29, 1073–1087. doi: 10.1105/tpc.16.00863. PMID: 28389585 PMC5466026

[B56] Zhang Z. Runions A. Mentink R. A. Kierzkowski D. Karady M. Hashemi B. . (2020). A WOX/Auxin biosynthesis module controls growth to shape leaf form. Curr. Biol. 30, 4857–4868.e4856. doi: 10.1016/j.cub.2020.09.037. PMID: 33035489

[B57] Zhou X. Han H. Chen J. Han H. (2024). The emerging roles of WOX genes in development and stress responses in woody plants. Plant Sci. 349, 112259. doi: 10.1016/j.plantsci.2024.112259. PMID: 39284515

[B58] Zhou S. Jiang W. Long F. Cheng S. Yang W. Zhao Y. . (2017). Rice homeodomain protein WOX11 recruits a histone acetyltransferase complex to establish programs of cell proliferation of crown root meristem. Plant Cell 29, 1088–1104. doi: 10.1105/tpc.16.00908. PMID: 28487409 PMC5466029

[B59] Zhou Y. Liu X. Engstrom E. M. Nimchuk Z. L. Pruneda-Paz J. L. Tarr P. T. . (2015). Control of plant stem cell function by conserved interacting transcriptional regulators. Nature 517, 377–U528. doi: 10.1038/nature13853. PMID: 25363783 PMC4297503

