# Supplementary material for: Genome-wide identification and characterization of WOX gene family in saffron (Crocus sativus L.) and their roles in stress response, development and callus formation
Source: Front Plant Sci. 2026 Mar 18;17:1764909. doi: 10.3389/fpls.2026.1764909 (PMC13038868; doi:10.3389/fpls.2026.1764909)
Supplement: Supplementary file 1 [file DataSheet1.docx]

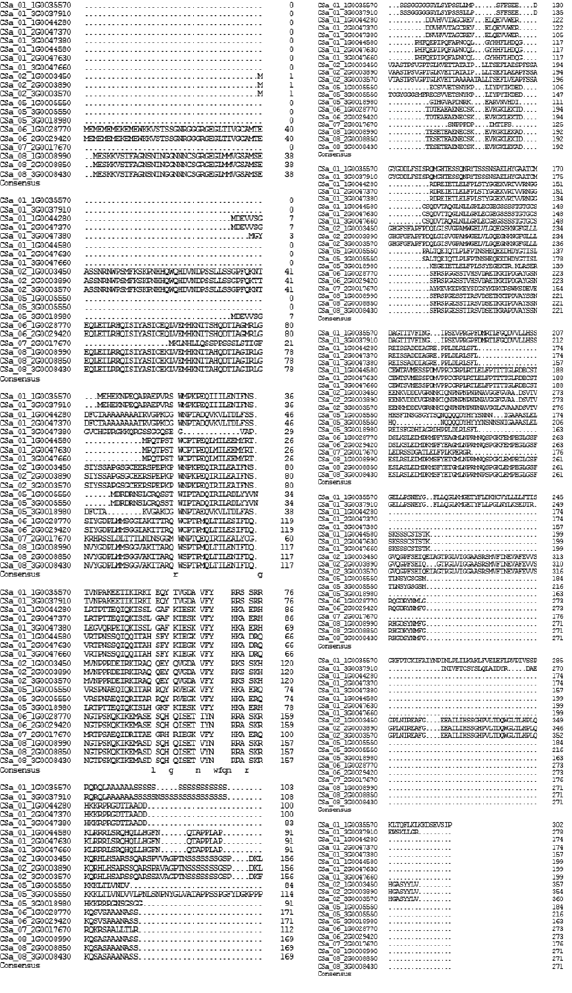


Supplementary Figure 1 Multiple sequence alignment of 20 *CsWOX* protein sequences.


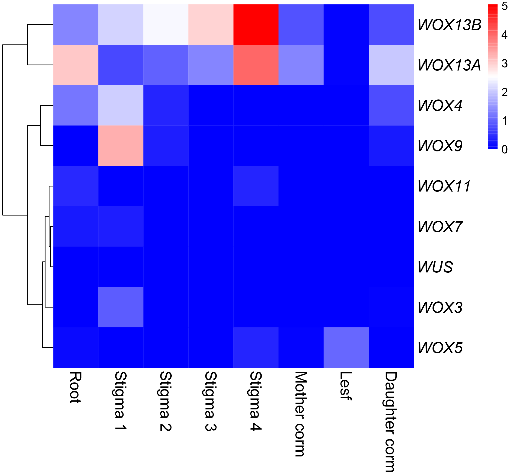


Supplementary Figure 2 *CsWOX* genes expression pattern in different stresses, flowering roots（Root），four stigma stages（Stigma1-Stigma4）and vegetative propagation stage tissues (Mother corm, Leaf and Daughter corm).


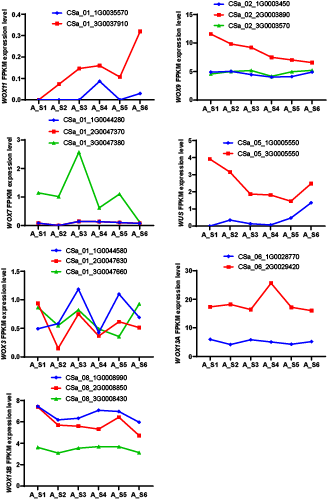


Supplementary Figure 3 Expression patterns of duplicated CsWOX genes in SAM with 22°C treatment.


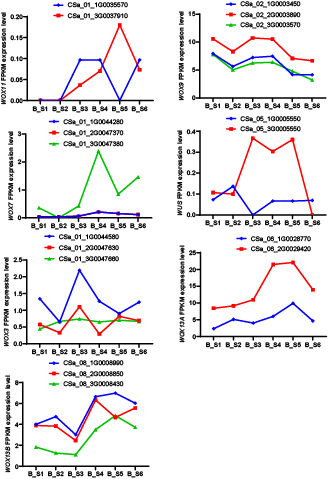


Supplementary Figure 4 Expression patterns of duplicated CsWOX genes in SAM with 9°C treatment.


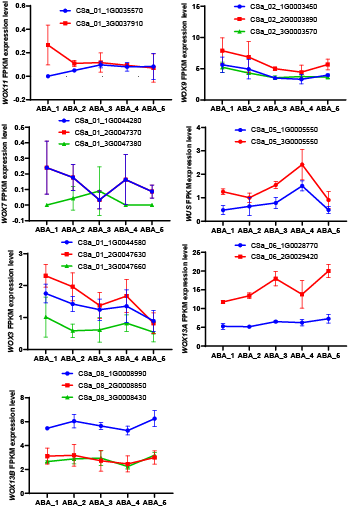


Supplementary Figure 5 Expression patterns of duplicated CsWOX genes in SAM with ABA treatment.


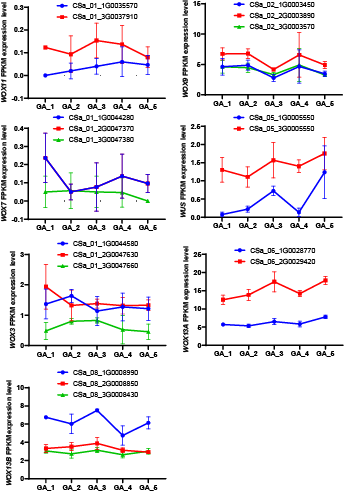


Supplementary Figure 6 Expression patterns of duplicated CsWOX genes in SAM with GA treatment.


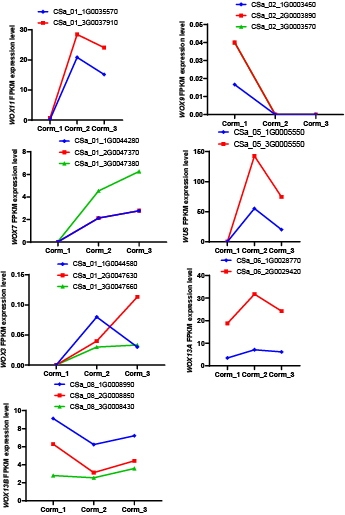


Supplementary Figure 7 Expression patterns of duplicated CsWOX genes with corm derived callus.


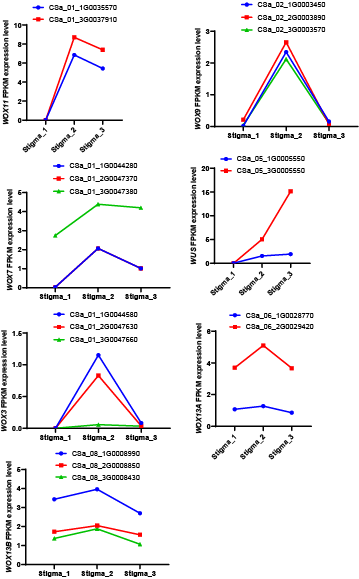


Supplementary Figure 8 Expression patterns of duplicated CsWOX genes with stigma derived callus.
